# Volvulus is Stressful: Stress-Induced Cardiomyopathy Secondary to Gastric Volvulus and Paraesophageal Hernia

**DOI:** 10.7759/cureus.61031

**Published:** 2024-05-24

**Authors:** Nadim Jaafar, Rahul Sharma, Jayashrei Sairam, Akshay Duddu

**Affiliations:** 1 Internal Medicine, Greater Baltimore Medical Center, Towson, USA

**Keywords:** critical care cardiology, paraesophageal hernia, organo-axial gastric volvulus, systolic heart failure, stress induced cardiomyopathy

## Abstract

Stress-induced cardiomyopathy (SCM) is a cardiac systolic dysfunction caused by various stressful triggers. It is often transient and reversible upon the reversal of the underlying stressor. We present the case of a 70-year-old female with SCM in the setting of gastric volvulus and incarcerated para-esophageal hernia.

## Introduction

Stress-induced cardiomyopathy (SCM) is a state of, often transient, cardiac systolic dysfunction driven by a myriad of stressful triggers. It is theorized to result from a catecholaminergic surge in the setting of physiological or psychological stress [1,2]. We present the case of a 70-year-old female with SCM in the setting of gastric volvulus and incarcerated para-esophageal hernia.

## Case presentation

A 70-year-old female with a past medical history of intellectual disability, hypertension, aortic calcification, and diabetes mellitus presented with acute onset abdominal pain and vomiting. The patient was unable to provide a detailed description of her presenting complaints. On admission, she was afebrile with a heart rate of 110 beats/minute, blood pressure of 136/86 mmHg, and respiratory rate of 16 breaths/minute. She was found to be hypoxic with oxygen saturation of 88% on room air; hence, she was placed on supplemental oxygen through a nasal cannula.

**Figure 1:**
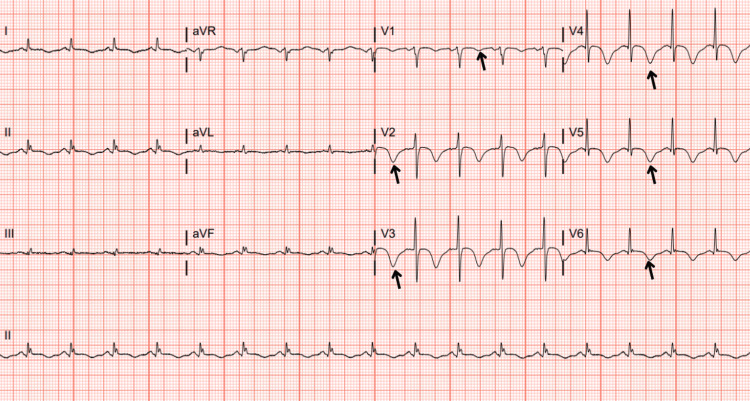
Electrocardiogram. Sinus tachycardia with a heart rate of 105 beats/minute and deep T-wave inversions in anterolateral leads (black arrows).

**Figure 2:**
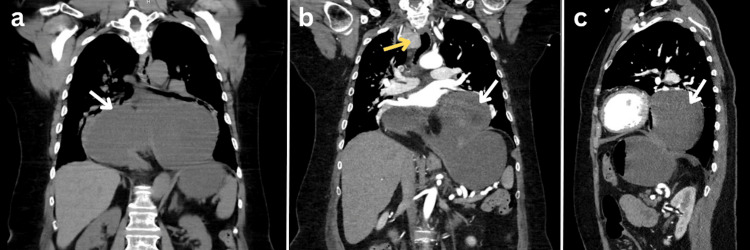
CT of chest, abdomen, and pelvis with and without contrast; (a and b) coronal view, (c) sagittal view Gastric obstruction secondary to a large hiatal hernia with a superimposed organo-axial gastric volvulus (white arrows) and enlarged thyroid with a mild right to left tracheal deviation (yellow arrow).

Initial labs revealed leukocytosis of 14.23x10³/µL, elevated lactic acid to 2 mmol/L, elevated troponin I at 1.230 ng/mL (peaked at 2.500 ng/mL), elevated pro-b-type natriuretic peptide (ProBNP) of 8,966 pg/mL, and a normal lipid panel. An EKG revealed sinus tachycardia with anterolateral deep T-wave inversions (Figure 1). A CT of the chest, abdomen, and pelvis with and without contrast revealed gastric obstruction secondary to a large hiatal hernia with a superimposed organo-axial gastric volvulus, enlarged thyroid with a mild resultant right to left tracheal deviation, and pleural effusion with possible pulmonary edema (Figure 2). A nasogastric tube was inserted and placed to low-intermittent suction, which resulted in symptomatic improvement.

**Figure 3:**
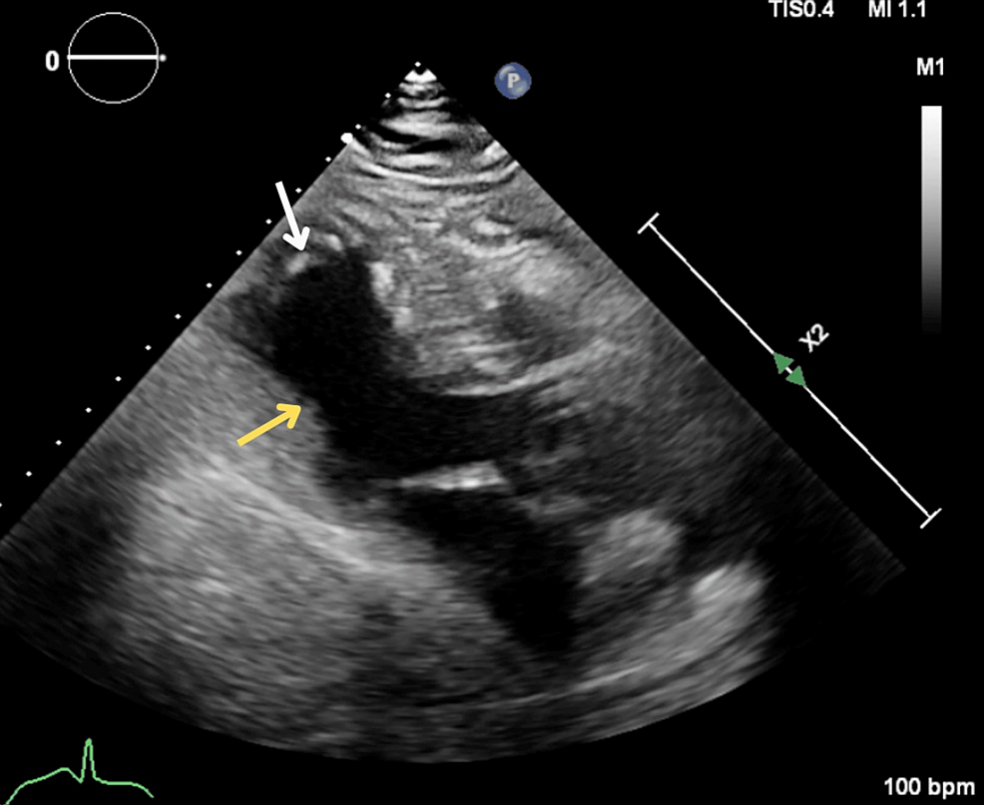
Initial echocardiogram. Parasternal long-axis view during systole, revealing concentric left ventricular hypertrophy with apical ballooning (white arrow) and preserved basal contractility (yellow arrow).

Given elevated troponin and ProBNP, and possible pulmonary edema on the chest CT, the patient underwent an echocardiogram that revealed concentric hypertrophy and an ejection fraction of 35-40% with apical ballooning and contractile cardiac bases (Figure 3), in addition to a grade-1 diastolic dysfunction. Cardiology was consulted, and while the aforementioned findings were suggestive of SCM, a non-ST elevation myocardial infarction could not be ruled out at that time. The patient was placed on a 48-hour continuous intravenous heparin drip and aspirin.

The patient was intubated and underwent an esophagogastroduodenoscopy, which revealed a large para-esophageal hernia. However, the scope could not pass beyond the stomach due to gastric outlet obstruction. The patient eventually underwent a laparoscopic reduction and fixation of the incarcerated para-esophageal hernia and gastric volvulus. Following the surgery, the patient was extubated and weaned off supplemental oxygen. She started participating in physical therapy with good resultant recovery.

An echocardiogram was repeated a week later, which revealed an improved ejection fraction of 45% with a hypokinetic apex but resolved apical ballooning. Furthermore, an echocardiogram a month later showed an ejection fraction of 50-55% with no regional wall abnormalities. In liaison with Cardiology, given the rapid improvement in echocardiographic findings, further ischemic workup was not pursued.

## Discussion

SCM, also known as Takotsubo cardiomyopathy, is a form of reversible systolic dysfunction that is brought on by stressful physiological or psychological triggers. It is theorized to be a result of catecholaminergic surge due to excessive sympathetic stimulation in states of stress [1,2]. It may mimic an acute coronary syndrome (ACS) as it involves left ventricular dysfunction, electrocardiographic changes, and elevations in cardiac biomarkers [3]. The alias Takotsubo cardiomyopathy, which translates to “octopus trap,” describes the left ventricular appearance on echocardiography and ventriculography of apical ballooning with preserved basal contraction [4]. However, other patterns, such as isolated basal and focal left ventricular wall dysfunction, have been described [5]. The true prevalence of SCM remains unknown; however, it has been estimated to be around 15-30 cases per 100,000 per year in the United States, with an overwhelming majority affecting females [6]. Management is typically through reversing the underlying stressor. While ischemic rule-out is often performed, our patient improved rapidly with the reversal of the stressor; thus, further testing was not pursued.

Moreover, gastric volvulus is a rare entity, mostly seen in pediatric patients and patients above 50 years of age with no predilection to gender. It occurs as the stomach twists around its longitudinal or transverse axes, compromising its blood flow [7]. Organoaxial rotation is the most common form of gastric volvulus, occurring as the stomach twists around the gastroesophageal junction and pylorus [8]. Additionally, gastric volvulus has been highly associated with para-esophageal hernias, as seen in our patient [9]. At early stages, gastric volvulus mortality ranges between 15% and 20%; however, mortality rates exponentially increase to 40-60% if the blood supply is severely compromised. Thus, early diagnosis and management are of utmost importance.

While a great physiological stress inducer, gastric volvulus is an exceedingly rare cause of SCM. Zawaideh et al. reported a case of mid-ventricular SCM in the setting of gastric volvulus due to diaphragmatic hernia, with complete resolution of cardiac function upon treating the volvulus [10]. To our knowledge, there have been no other reports associating SCM with gastric volvulus. However, there have been reports of gastric volvulus inducing EKG changes [11], chest pain [12], and arrhythmias (supraventricular tachycardia) [13].

## Conclusions

SCM is often a transient cardiac systolic dysfunction caused by physical or psychological stressors. It may mimic ACS in symptoms, EKG changes, cardiac biomarkers, and/or echocardiographic findings. One of the most prevalent echocardiographic patterns is left ventricular apical dilatation with preserved basal contractility. We presented a rare case of SCM occurring in the context of gastric volvulus and incarcerated para-esophageal hernia.
